# Changes in the Leaf Physiological Characteristics and Tissue-Specific Distribution of Ginsenosides in *Panax ginseng* During Flowering Stage Under Cold Stress

**DOI:** 10.3389/fbioe.2021.637324

**Published:** 2021-03-17

**Authors:** Tao Zhang, Changbao Chen, Yuqiu Chen, Qinghe Zhang, Qiong Li, Weichen Qi

**Affiliations:** Key Laboratory of Chinese Medicine Planting and Development, Changchun University of Chinese Medicine, Changchun, China

**Keywords:** *Panax ginseng*, biomass, cold stress, biotechnology, ginsenoside

## Abstract

*Panax ginseng* is a valuable traditional herbal medicine material with numerous applications. Ginsenosides are the key bioactive compounds in ginseng. Cold stress can activate stress tolerance mechanisms that regulate biomass and biosynthesis in ginseng tissue. In this study, the effects of short- and long-term cold stress (5°C) on the physiological characteristics, tissue-specific ginsenoside distributions, and ginsenoside synthesis gene expressions of 3-year-old *P. ginseng* during the flowering period were investigated. Short-term cold stress significantly reduced ginseng biomass (root fresh weight and dry weight), and increased malondialdehyde, proline, soluble sugar, and soluble protein concentrations. Superoxide dismutase, peroxidase, and catalase activities also increased significantly under cold stress. With prolongation of the cold stress period, all antioxidant enzyme activity decreased. The protopanaxatriol-type ginsenoside concentrations in the taproots (phloem and xylem) and fibrous roots, as well as the protopanaxadiol-type ginsenoside concentrations in the leaves, increased significantly under short-term cold stress. The key genes (*SE*, *DS-II*, *CYP716A52v2*, and *CYP716A53v2*) involved in the ginsenoside biosynthesis pathway were significantly positively correlated with the ginsenoside accumulation trends. Thus, short-term cold stress can stimulate membrane lipid peroxidation, in turn stimulating the antioxidant enzyme system to alleviate oxidative damage and increasing the expression of key enzyme genes involved in ginsenoside biosynthesis. During agricultural production, protopanaxadiol/protopanaxatriol ratios could be manipulated by low-temperature storage or treatments.

## Introduction

*Panax ginseng* C.A. Meyer is a perennial herb and a valuable traditional Chinese medicinal material with a long history of exploitation. Ginsenosides are the key bioactive compounds in ginseng. Modern pharmacological studies have demonstrated that ginsenoside has numerous medicinal applications, such as immunity regulation, anti-stress, anti-fatigue, anti-oxidation, anti-inflammatory, anti-tumor, hypoglycemic, and liver-protective properties (Chao, 2006; Leung and Wong, 2010; Lian-Wen et al., 2011; Liu, 2012). Currently, approximately 180 types of ginsenoside monomers have been isolated from ginseng (Christensen, 2009; Sun et al., 2011; In-Ah et al., 2012; Shi et al., 2013).

Ginsenosides are considered critical biomarkers in ginseng quality evaluation. The 2020 edition of the Chinese Pharmacopeia defines the appropriate amounts of root ginsenosides: the total Rg1 (C_42_H_72_O_14_) and Re (C_48_H_82_O_18_) content should not be lower than 0.3% and the Rb1 (C_54_H_92_O_23_) content should not be lower than 0.2%. The various ginsenoside aglycones can be divided into oleanane-type pentacyclic triterpene saponins, protopanaxadiol-type saponins (PPD), and protopanaxatriol-type saponins (PPT); both the PPD-type and PPT-type saponins are dammarane-type tetracyclic triterpenoids (Li et al., 2009).

To date, numerous studies have investigated the relationships between ginsenoside composition and biological activity. One study confirmed a close relationship between the biological activity of ginseng and the year of cultivation (Shan et al., 2014). Changes in the proportions of different ginsenosides are a major factor influencing changes in the biological activity of ginseng over time. The PPD/PPT ratio is considered a key factor influencing variations in the biological activity of ginseng (Cho et al., 2017). PPD- and PPT-type ginsenosides have different biological functions, and differences in their composition strongly influence the quality of medicinal materials (Qu et al., 2009).

Cold stress, a major abiotic stress factor, causes plants to adopt strategies that alter their external morphology, and the structure and accumulation of their secondary metabolites (Kim et al., 2013). In addition, a series of physiological and biochemical changes occur in the plant to counteract the potential damage caused by cold stress. Malondialdehyde (MDA) is a critical indicator of the degree of membrane peroxidation. Therefore, MDA concentration can directly reflect the degree of cell membrane damage (Lukatkin et al., 2012). Under stressful environmental conditions, the cell membrane system transmits signals to the plant and the reactive oxygen species (ROS) concentrations in the plant increase significantly. This represents the plants’ antioxidant enzyme systems facilitating resistance against adverse environmental factors and maintaining the integrity and stability of the cell membrane. Studies have demonstrated that cold stress can promote the production of antioxidant enzymes such as catalase (CAT), peroxidase (POD), and superoxide dismutase (SOD) in plant tissues, in addition to inducing increases in the accumulation of osmotic regulatory substances, such as proline (Pro), soluble sugar (SS), and soluble protein (SP). Short-term cold stress increases antioxidant enzyme activity, and increases the synthesis of osmotic regulators; however, under long-term cold stress, antioxidant enzyme activity is inhibited, and the concentrations of osmotic regulators decrease (Lo’Ay and Elkhateeb, 2018; Rezaie et al., 2020).

The biosynthesis of ginsenosides, the primary active compounds in ginseng, is influenced by interactions between genetic and environmental factors. The biosynthetic pathway of ginsenosides is divided into three parts: the main upstream genes include 3-hydroxy-3-methylglutaryl-CoA reductase (*HMGR*), farnesyl pyrophosphate synthase (*FPS*), squalene synthetase (*SS1*), squalene epoxidase (*SE*), dammarenediol synthase (*DS*), and aromatase (*PNY*) are the main genes included in the synthesis section; and cytochrome P450 (CYP450) and UDP-glycosyltransferase (UGT) are the main genes downstream of synthesis (Ohyama et al., 2007; Han et al., 2010; Wu et al., 2012; Yang et al., 2018). Studies have demonstrated that temperature could influence the expression of key enzyme genes in the ginsenoside synthesis pathway, and, in turn, the concentrations of ginsenosides (You et al., 2015; Jiang et al., 2016).

In the present study, the physiological indicators of ginseng resistance to abiotic stress were systematically analyzed, including antioxidant enzymes, osmotic regulator molecules, and secondary metabolism (key enzyme gene expression and secondary metabolites). The results of the present study should clarify the potential mechanisms of the physiological and biochemical responses of ginseng to cold stress and facilitate attempts to increase ginsenoside concentrations during ginseng cultivation.

## Materials and Methods

### Plant Materials and Experimental Design

The plant materials used were 3-year-old *P. ginseng* that were purchased from Fusong County, Jilin Province, China, and potted at the end of April 2018. The pots were 16.00 cm tall, with rim diameters of 23.00 cm and base diameters of 13.00 cm. The soil depth was 13.00 cm. Each pot was buried entirely in the soil and the temperature in the pot was maintained at the temperature of the surrounding soil. The soil water content was maintained at 60% of the field water holding capacity. The test soil was microbial substrate soil (44 bags of microbial substrate: 3 bags of vermiculite: 7 bags of perlite). There were three plants per pot, which were regularly watered, weeded, fertilized, and sprayed with pesticides. The flowering stage is a critical period for the growth and development of ginseng, especially for the reproductive organs. Studies have shown that the content of ginsenosides varies greatly during the flowering stage, which has important research significance (Yang et al., 2017). Therefore, in this study, plants in good condition were selected during the flowering stage (June 14–28) for use in the cold stress experiments. The water and light conditions were controlled according to the general requirements under field cultivation, and the day and night lengths were not changed.

A digital artificial climate room was adopted for carrying out the cold stress experiments (5°C). Sampling began on the first day of treatment and was carried out daily. Control (CK) treatments were set up in the field. During sampling, nine plants were sampled in each group, and the collected ginseng were placed in an ice box and transported to the laboratory. In the laboratory, sediment was washed off with running water, and the surface moisture was absorbed using filter paper. Afterward, the fresh weight was determined and recorded, and the fibrous roots, phloem, xylem, rhizomes, and leaves were separated. An appropriate number of leaves was obtained, placed in a self-sealing bag, and stored in liquid nitrogen at −80°C for later physiological tests. Tissues were ground into fine powder and sieved through a 60-μm mesh sieve for use in the determination of ginsenoside content.

### Reagents and Standards

The ginsenoside standards, Ro, Rg1, Re, Rf, Rb1, Rc, Rb2, Rb3, Rd, Rh2, and Rg3, were purchased from the National Institute for the Control of Pharmaceutical and Biological Products (Beijing, China). To satisfy the ultra-performance liquid chromatography analysis requirements, the purity of all standards was greater than 98%. High-performance liquid chromatography (HPLC)-grade acetonitrile and methanol were purchased from Thermo Fisher Scientific (Norcross, GA, United States), and the HPLC water used was Wahaha mineral water (Jilin, China). SOD, POD, CAT, SP, Pro, SS, and MDA assay kits were purchased from the Nanjing Jiancheng Bioengineering Institute (NJBI, Nanjing, China).

### Sample and Standard Solution Preparation

A ginseng powder sample (1.0 g) was placed in an Erlenmeyer flask and extracted according to the pharmacopeia method, with slight modification (Kuang et al., 2020). Afterward, 30 mL of methanol was added, and extracted by ultrasonication at 90 Hz for 30 min. The supernatant was then filtered, and the procedure repeated three times. The filtrates were combined and transferred to an evaporating dish and evaporated in a 60°C water bath. The volume was brought to 5 mL in a volumetric flask with a 0.22-μm pinhole filter for HPLC analysis (Guo et al., 2015). Measurements were performed in triplicate.

To establish the calibration curves of the 11 ginsenoside standards (Ro, Rg1, Re, Rf, Rb1, Rc, Rb2, Rb3, Rd, Rh2, and Rg3), stock solutions containing the 11 standards were weighed precisely and dissolved in methanol. Then, the solutions were diluted into a series of standard solutions with gradient concentrations. The solutions were passed through a 0.22-μm membrane and stored in a refrigerator at 4°C.

### HPLC Analytical Conditions

The prepared samples were analyzed using an Agilent 1260 HPLC system (Agilent, United States) and separated in an Elite Hypersil ODS2 (250 mm × 4.6 mm, 5 μm) column. The mobile phase was composed of A (water) and B (acetonitrile). The gradient elution was performed as follows: 0–18.0 min, 19–23% B; 18.1–28.0 min, 23–28% B; 28.1–30.0 min, 28–32% B; 30.1–50.0 min, 32–34% B; and 50.1–70.0 min, 34–80% B. The injection volume was 10 μL, the column temperature was 25°C, UV measurements were obtained at 203 nm, and the flow rate was 1.0 mL/min.

### Determination of Osmoregulatory Substances and Malondialdehyde Content

In an ice bath, 0.5-g fresh root tissues were homogenized (by grinding) in 5 mL of phosphate-buffered saline (0.05 mol’L^–1^, pH 7.8). The homogenates of the fresh root tissues were assayed with SP, Pro, SS, and MDA kits (NJBI). The absorbance of the reaction solutions was measured at 595, 520, 620, and 530 nm using an enzyme-labeling instrument (SpectraMax 190, Molecular Devices, United States); the values obtained were used to calculate the concentrations of SP, Pro, SS, and MDA, respectively.

### Antioxidant Enzyme Extraction and Determination of Enzyme Activity

The method of crude enzyme extract preparation used in the present study was modified from a previously published method (Ba et al., 2013). The samples were ground into homogenates and transferred into centrifuge tubes; afterward, phosphate-buffered saline (0.05 mol’L^–1^, pH 7.8) was added up to final volumes of 5 mL. The samples were then centrifuged at 4°C and 10000 rpm’min^–1^ for 10 min. SOD, POD, and CAT activities were assayed using kits (NJBI) and their activities were expressed as units per milligram of protein.

### Extraction of RNA and Gene Expression of Key Enzymes

Total ginseng RNA was extracted using a TaKaRa MiniBEST Universal RNA Extraction Kit (TaKaRa, CA, Japan) and 1.0 μg RNA was used for reverse transcription with a PrimeScript^TM^ RT Master Mix kit (TaKaRa, CA, Japan) in a 20-μL reaction volume, according to the manufacturer’s instructions. The product was stored at −20°C. GAPDH was used as an internal control, and the relative gene expression levels of *HMGR2*, *FPS*, *SS1*, *SE1*, *DS-II*, *PNY1*, *CYP716A52v2*, *CYP716A53v2*, and *CYP716A47* were determined (primer information is listed in Table 1). Reverse transcriptase quantitative PCR (RT-qPCR) was performed in 96-well plates in a Stratagene Mx3000P thermocycler (Agilent, Palo Alto, CA, United States) with an SYBR Green-based PCR assay. The final volume for each reaction was 20 μL with the following components: 1 μL diluted cDNA template (1 mg/mL), 10 μL SYBR Green Mix (TaKaRa, DaLian, CA, Japan), 1 μL forward primer (1 mM), 1 μL reverse primer (1 mM), and 7 μL ddH_2_O. The reaction was performed under the following conditions: 95°C for 3 min, followed by 40 cycles of denaturation at 95°C for 5 s, annealing at 55°C for 32 s, and extension at 72°C for 20 s. The melting curve was obtained by heating the amplicon from 55 to 95°C with increments of 0.5°C per 5 s. Each RT-qPCR analysis was performed with three biological replicates. The relative gene expression levels were computed using the 2^–Δ^ ^Δ^ ^Ct^ method.

**TABLE 1:** Real-time fluorescence quantitative PCR primers.

Gene	Accession No.	Primer sequence 5′-3′	Product length (bp)
*GAPDH*	KY400031	F: ATGGACCATCAGCAAAGGAC R: GGTAGCACTTTCCCAACAGC	117
*HMGR2*	JX648390	F: TCTTCAAAGCCTCTGATGC R: TTTTGGGGATTGGATTTGTCA	126
*FPS*	DQ087959	F: CAAGAAGCATTTCCGACAA R: CTCTCCTACAAGGGTGGTGA	116
*SS1*	AB115496	F: GGACTTGTTGGATTAGGGTTG R: ACTGCCTTGGCTGAGTTTTC	107
*SE1*	AB122078	F: ATGCTTTGAATATGCGCCATC R: CATGGAGATCGCGTAAAGGTC	102
*DS-II*	AB265170	F: CAAATGCCACAAGGATATTGTC R: TGCGAAACCACCACTTACAC	105
*PNY1*	AB009030	F: GCGGAAGGGAATAAGATGAC R: CTCAGCTCTCCGGACAGC	108
*CYP716A47*	JN604536	F: TCACCTTCGTTCTCAACTATC R: TCTTCCTCAAATCCTCCCAAT	129
*CYP716A52v2*	JX036032	F: AGGAGCAAATGGAGATAG R: AACCGTTGTAGGTGAAAT	106
*CYP716A53v2*	JX036031	F: ATCGGACAACGAGGCAGCAC R: GCCAACAGGCCAACTCAA	102

### Statistical Analysis

MS Excel 2016 (Microsoft Corp., Redmond, WA, United States) was used to sort the original data, and IBM SPSS Statistics 19.0 (IBM Corp., Armonk, NY, United States) was used for single-factor variance analysis. GraphPad prism 6.0 (GraphPad Software Inc., San Diego, CA, United States) and Origin 9.0 (OriginLab, Northampton, MA, United States) were used for graphic illustration.

## Results

### Root Fresh and Dry Weight of *Panax ginseng* Under Cold Stress

**FIGURE 1:**
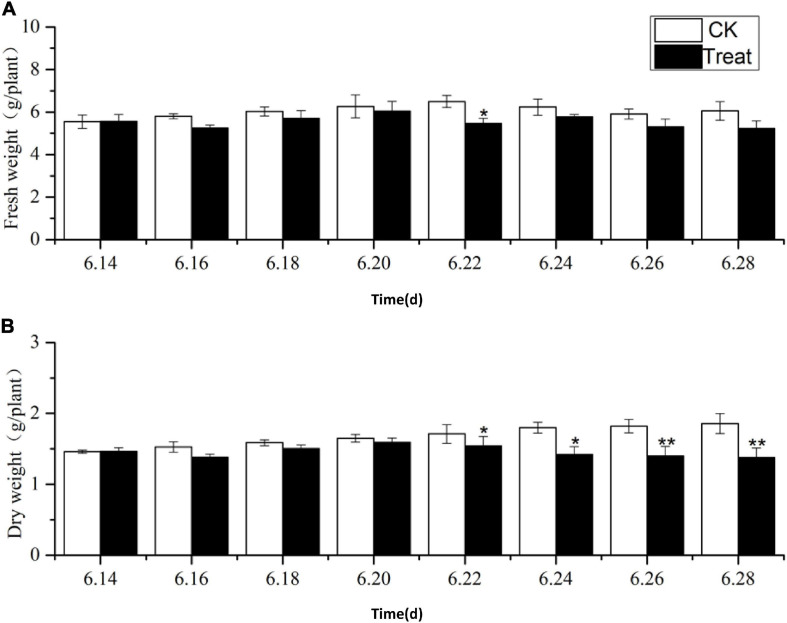
Effects of cold stress on fresh weight and dry weight of ginseng during the flowering stage. The *X* axis represents the sampling time. **(A)** Represents the change of fresh weight under cold stress. **(B)** Represents the change of dry weight under cold stress. *p < 0.05 and **p < 0.01, compared with control group. Vertical bars indicate the mean value ± standard deviation from three independent experiments. The same below.

The effects of cold stress on ginseng root fresh and dry weight at the flowering stage are illustrated in Figure 1. During the entire temperature treatment stage, the fresh and dry weights of the roots increased to varying degrees, and the trends in the root dry and fresh weights were similar from June 22 to 28. The root fresh and dry weights in the treatment groups were lower than those in the control group, and they dropped significantly on June 22 and 26 by 15.8 and 23.1%, respectively. Long-term cold stress treatment induced a series of changes in the physiological and metabolic processes of ginseng, which, in turn, inhibited the accumulation of dry and fresh weight in the ginseng roots.

### Ginsenoside Concentration Detection and Method Establishment

**FIGURE 2:**
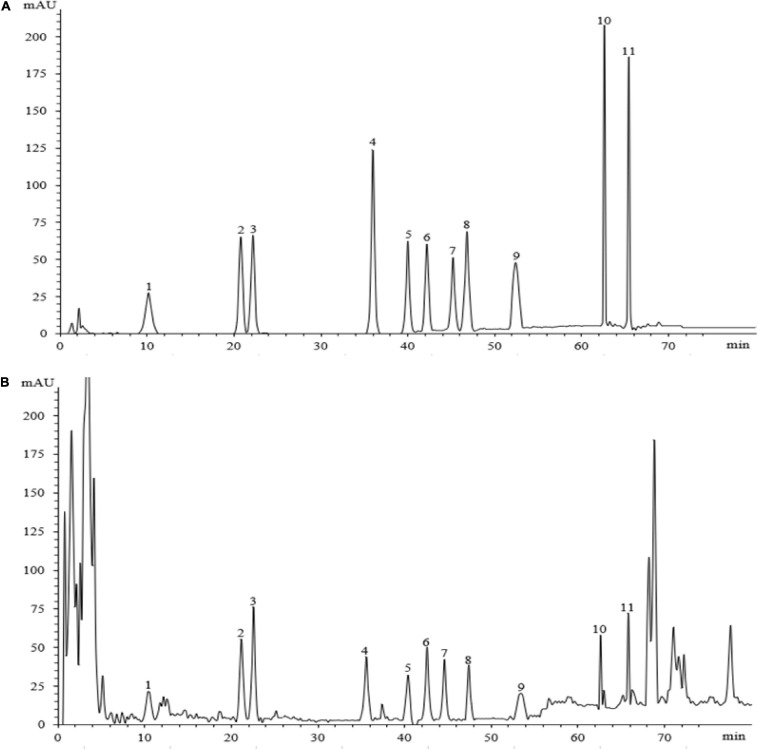
The HPLC chromatograms of the standard and sample. Panel **(A)** shows the chromatographic peak of the standard substance, panel **(B)** shows the chromatographic peak of the ginseng root sample. The peaks 1–11 represent ginsenosides-Ro, -Rg1, -Re, -Rf, -Rb1, -Rc, -Rb2, -Rb3, -Rd, -Rh2, and -Rg3.

Eleven ginsenosides were identified based on the retention times of each saponin monomer peak. Figure 2 presents the HPLC spectrum of the standards (Figure 2A) and the main root extract (Figure 2B). Linear regression equations were obtained by taking the peak area as the ordinate and the concentration of the reference solution as the abscissa. The 11 compounds exhibited good linear relationships within their respective linear ranges. The results are listed in Table 2. The 11 compounds were detected in the main root of the ginseng. The 11 detected constituents had good resolution and could be easily determined. They were also the primary constituents in the assayed samples. The established HPLC quantitative method had good linear ranges and was consistent with the relative requirements.

**TABLE 2:** Linear regression equations, correlation coefficients, and linearity ranges of 11 ginsenosides.

Ginsenosides	Regression Equation	*R*^2^	Linear Range (μg/mL)
Ro	*y* = 2796.01 x - 40.64132	0.99969	0.03–0.40
Rg1	*y* = 3131.42 x - 34.75556	0.99943	0.01–0.35
Re	*y* = 3097.80 x - 35.99583	0.99945	0.04–0.50
Rf	*y* = 3614.44 x - 32.36528	0.99912	0.01–0.40
Rb1	*y* = 2246.85 x - 17.05278	0.99989	0.01–0.20
Rc	*y* = 2528.59 x - 19.43056	0.99985	0.03–0.40
Rb2	*y* = 2668.61 x - 36.21944	0.99941	0.01–0.20
Rb3	*y* = 3451.35 x - 47.50556	0.99932	0.01–0.20
Rd	*y* = 3009.68 x - 30.33472	0.99967	0.02–0.30
Rh2	*y* = 3813.16 x - 23.14722	0.99946	0.01–0.10
Rg3	*y* = 4742.04 x - 45.57500	0.99955	0.01–0.30

### Change of Ginsenoside Content in Different Ginseng Tissues During the Flowering Stage Under Cold Stress

#### Change of Rg1 Content

**FIGURE 3:**
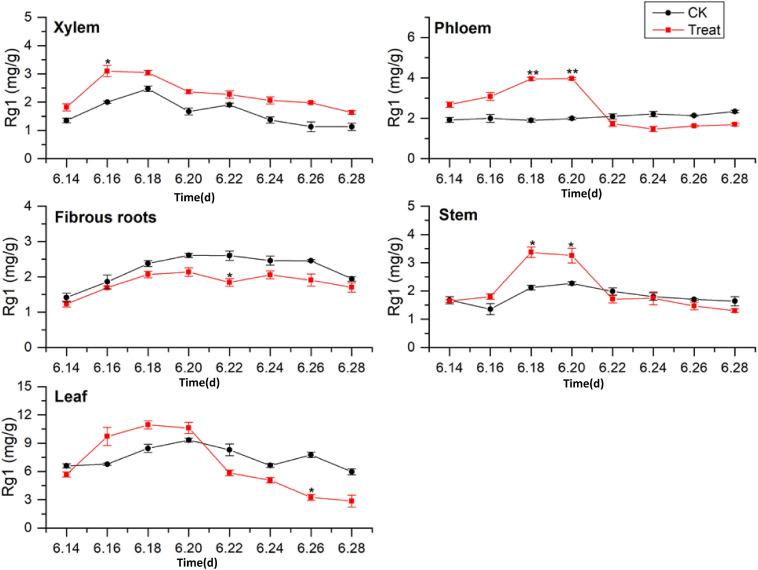
Effects of cold stress on Rg1 in different tissues of flowering ginseng. The *X* axis represents the sampling time. *p < 0.05 and **p < 0.01, compared with control group. The data are expressed as the mean ± SD (*n* = 3).

As illustrated in Figure 3, the tissue specificity of flowering ginseng was determined by analyzing the concentrations of Rg1 in each of the tissues in the control group. The order of Rg1 concentration was leaf > fibrous roots > phloem > stem > xylem. The leaf was the primary synthetic site of Rg1, which is consistent with the findings of Kim et al. (2015).

After cold stress treatment, the concentrations of Rg1 in xylem, phloem, stem, and leaf all exhibited increasing trends, followed by decreases. The concentrations of Rg1 in the xylem, phloem, stem, and leaf all increased to varying degrees, and the peaks were observed on June 16, 20, 18, and 18, with concentrations 54.5, 98.7, 58.9, and 29.9% higher than those in the control group, respectively. The xylem Rg1 concentrations were higher than those of the control group throughout the flowering period. The leaf Rg1 concentrations decreased from June 22 to levels below those of the control group, while the fibrous root Rg1 concentrations were suppressed throughout the treatment period following cold stress to levels lower than those in the control group. According to the time taken for a significant difference in the Rg1 content to appear, the order of sensitivity to cold stress was phloem > stem > xylem > leaves > fibrous roots.

#### Change of Rb1 Content

**FIGURE 4:**
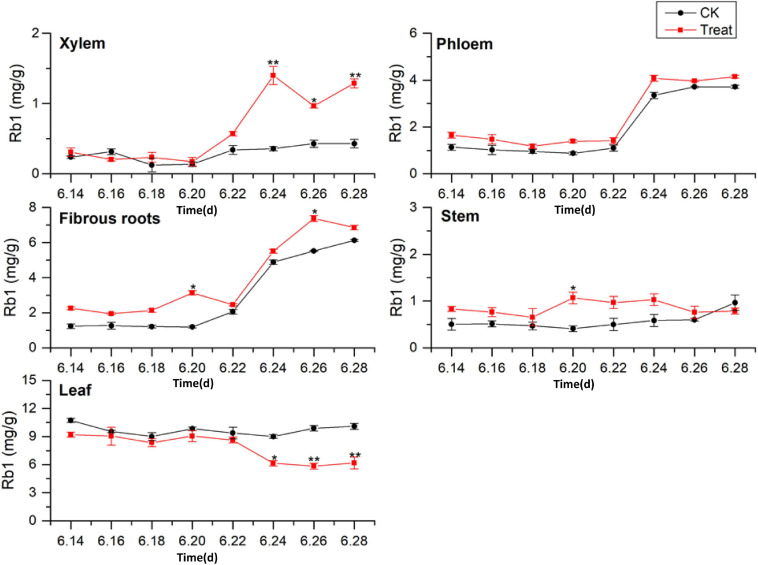
Effects of cold stress on Rb1 in different tissues of flowering ginseng. The *X* axis represents the sampling time. *p < 0.05 and **p < 0.01, compared with control group. The data are expressed as the mean ± SD (*n* = 3).

The tissue-specific distributions of ginsenosides in flowering ginseng were analyzed based on the Rb1 concentrations in each tissue in the control group (Figure 4). The order of Rb1 concentrations was leaf > fibrous roots > phloem > stem > xylem, indicating that the leaf is also a primary site of Rb1 biosynthesis during flowering. The concentrations of Rb1 in the fibrous roots and phloem, both after cold stress treatment and in the control group, exhibited gradual upward trends and began to rise rapidly on June 22. Although cold stress seemed to promote the accumulation of phloem Rb1, its effect was not significant. Cold stress promoted the accumulation of Rb1 in the fibrous roots on June 20 and 26 (162.4 and 33.5% higher than that in the control group, respectively).

The concentrations of Rb1 in the treated phloem and stem also seemed to be higher than those in the control group, but there was no significant difference. The concentration in the treated xylem increased rapidly on June 22, and was significantly different from the concentration in the control group; by June 24, the treated xylem Rb1 concentration had increased by 288.8% compared with the concentration in the control group. The Rb1 concentrations in the treated stem were significantly different from the concentrations in the control group on June 20, representing a 145.5% increase. The Rb1 concentrations in the leaves were higher than those in other tissues, with relatively stable levels. Following cold stress treatment, the leaf Rb1 content decreased, and the decrease became significant on June 26, when it was 37.3% lower than the level in the control group. Rb1 biosynthesis in the xylem, phloem, fibrous roots, and stem were promoted, but Rb1 biosynthesis in the leaves was inhibited. The Rb1 biosynthesis trends under cold stress in different tissues was xylem > fibrous roots > stem > leaf > phloem.

#### Change of Re Content

**FIGURE 5:**
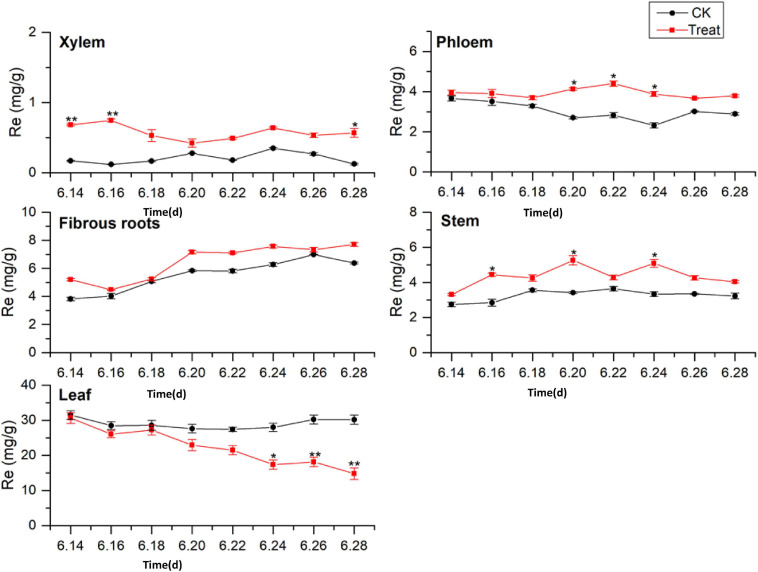
Effects of cold stress on Re in different tissues of flowering ginseng. The *X* axis represents the sampling time. *p < 0.05 and **p < 0.01, compared with control group. The data are expressed as the mean ± SD (*n* = 3).

Ginsenoside tissue specificity was determined by analyzing the content of Re in different tissues (Figure 5). The Re concentrations in the tissues were in the order leaf > fibrous roots > phloem > stem > xylem. The Re concentrations in the leaves were 3 to 5-fold higher than those in the fibrous roots, indicating that the leaves were the primary Re biosynthesis sites during flowering. The Re content in the fibrous roots exhibited a gradual upward trend, while the Re content in the xylem, phloem, stem, and leaf remained stable, with no significant changes during flowering. After cold stress treatment, the Re content increased to varying degrees and remained higher than the content in the control group throughout the flowering period. The treated xylem achieved its maximum Re value on June 16, 243.3% higher than the content in the control group, whereas the treated phloem achieved its maximum value on June 22, which was 170.7% higher than the content in the control group.

The content of Re in the treated fibrous roots and stems reached their highest levels on June 20, 23.2 and 53.9% higher than those in the control group, respectively. The Re content in the leaves under cold stress was lower than that in the control group. On June 24, it was significantly lower than that in the control group, and it fell to the lowest point on June 28, 50.8% lower than the control group. Cold stress promoted the biosynthesis of Re in the xylem, phloem, fibrous roots, and stems, and inhibited the biosynthesis of Re in the leaves. According to the time taken for a significant difference in the Re content to appear, the order of sensitivity to cold stress was xylem > stem > phloem > leaves > fibrous roots.

#### Change of Total Saponin Content

**FIGURE 6:**
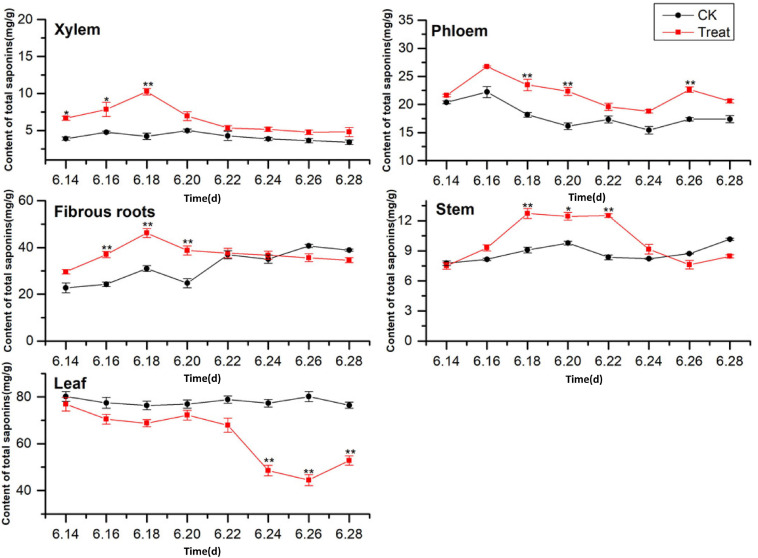
Effects of cold stress on total saponin content in different tissues of flowering ginseng. The *X* axis represents the sampling time. *p < 0.05 and **p < 0.01, compared with control group. The data are expressed as the mean ± SD (*n* = 3).

A comparison of the total saponin content in different ginseng tissues under cold stress treatment revealed that the total saponin content in the control tissues was ordered leaf > fibrous roots > phloem > stem > xylem, and cold stress increased the total saponin content in the xylem, phloem, fibrous roots, and stem (Figure 6). The total saponin content in the xylem was relatively low and did not change significantly across the flowering period. After cold stress treatment, the total saponin content in the xylem first increased and then decreased. It was higher than that in the control group throughout the flowering period, and reached its highest value on June 18, when it displayed a 143.5% increase. Gradual decreasing trends were exhibited by the phloem. The trend following cold treatment was similar to that in the control group; however, the total saponin content was higher than that in the control group, with significant differences on June 18, 20, and 26.

The total saponin content in the fibrous roots and stems in the control groups exhibited gradual upward trends. After cold stress treatment, the total saponin content increased significantly followed by downward trends. The maximum values for the treated fibrous roots and stems were observed on June 18 (48.89 and 40.1% higher than the control values, respectively). The total saponin content in the control leaves was the highest of all the tissue samples, and there was no significant change during the flowering period. The concentrations were lower than the control group under cold stress, with the minimum value observed on June 26, 44.3% lower than the control value. These results indicate that cold stress promoted the accumulation of saponins in the xylem and phloem and inhibited the accumulation of total saponins in the leaves. In addition, short-term cold stress promoted the accumulation of saponins in the fibrous roots and stems. The synthesis and accumulation of total saponins in different tissues in response to cold stress followed the order xylem > fibrous roots > leaf > stem > phloem.

#### Change of PPD Content

**FIGURE 7:**
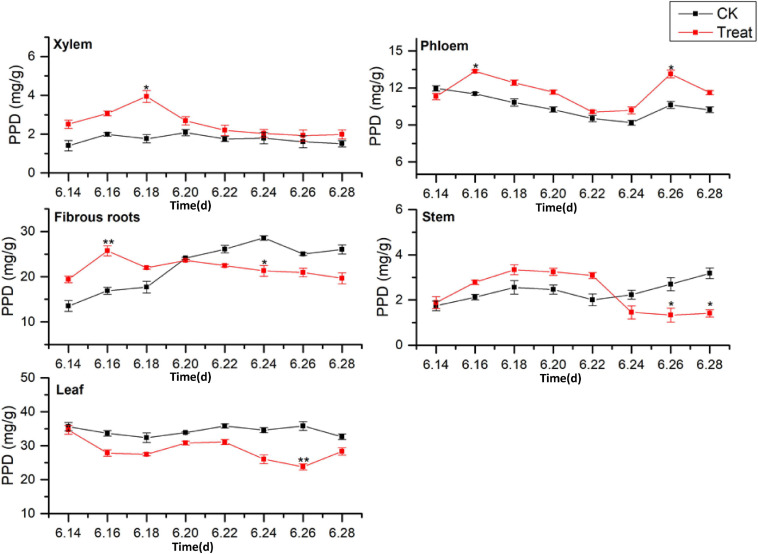
Effects of cold stress on the PPD ginsenoside content in different tissues of flowering ginseng. The *X* axis represents the sampling time. *p < 0.05 and **p < 0.01, compared with control group. The data are expressed as the mean ± SD (*n* = 3).

Dammarane-type ginsenosides are divided into PPD- and PPT-type ginsenosides, according to their glycosyl groups. The effects of cold stress on the accumulation of PPD-type ginsenosides (PPD = Rb1 + Rb2 + Rc + Rd + Rb3 + Rh2 + Rg3) in different ginseng tissues are illustrated in Figure 7. The PPD-type ginsenoside concentrations in the control groups followed the order leaf > fibrous roots > phloem > stem > xylem, similar to the total saponin content trends. The PPD ginsenoside content in the xylem was low. Under cold stress, the xylem PPD concentrations accumulated rapidly to the maximum value, which was observed on June 18 and was 123.8% higher than the value in the control group, but the effect was short-lived. The phloem PPD ginsenoside concentrations exhibited declining trends followed by rising trends. After cold stress treatment, the maximum values were observed on June 26, 23.6% higher than the levels in the control group.

The PPD ginsenoside concentrations in the control fibrous roots exhibited gradual increasing trends. After cold stress, the concentrations first increased and then decreased. The maximum values were observed on June 16, displaying a 52.5% increase compared to the levels in the control group. They then decreased to levels below the levels in the control group on June 20. The PPD ginsenoside concentrations in the control stems slowly increased, then decreased, and finally rose rapidly. After cold stress treatment, they first increased and then decreased, with the minimum value observed on June 26, 50.6% lower than the level in the control group. The PPD ginsenoside content in the control leaves was the highest of all the tissue samples and remained relatively stable. After cold stress treatment, the content was lower than that in the control group, and reached a minimum on June 26, 33.6% lower than the content in the control group. These results indicate that cold stress promoted the accumulation of PPD ginsenosides in the xylem and phloem and inhibited their accumulation in the leaves, while short-term cold stress promoted PPD ginsenoside accumulation in the fibrous roots and stems. This is the same as the pattern shown by the total saponins responding to the cold treatment. The order of the PPD ginsenoside synthesis and accumulation rates in different tissues in response to cold stress was xylem > fibrous roots > stem > leaf > phloem.

#### Change of PPT Content

**FIGURE 8:**
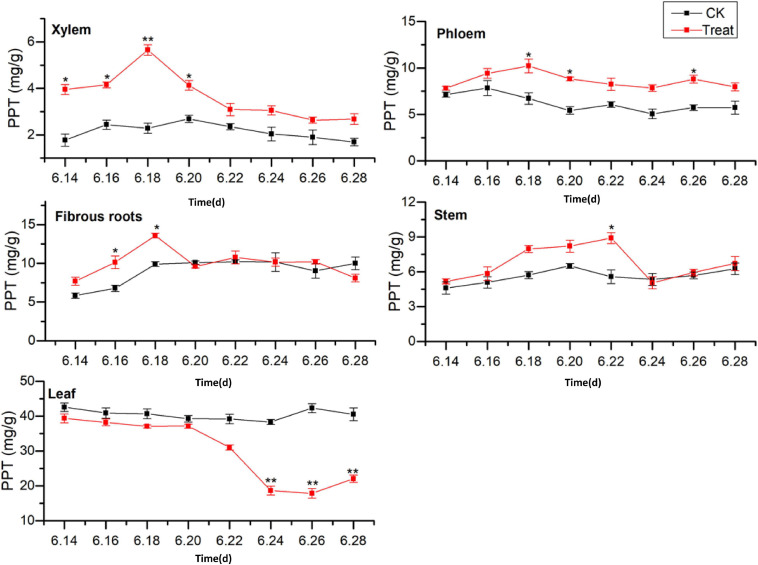
Effects of cold stress on the PPT ginsenoside content in different tissues of flowering ginseng. The *X* axis represents the sampling time. *p < 0.05 and **p < 0.01, compared with control group. The data are expressed as the mean ± SD (*n* = 3).

The effects of cold stress on the accumulation of PPT-type ginsenosides (PPT = Re + Rg1 + Rf) in different ginseng tissues during flowering are illustrated in Figure 8. The PPT-type ginsenoside content of the different tissues was ordered leaf > fibrous roots > phloem > stem > xylem, which is similar to the accumulation patterns of the total saponins and PPD-type ginsenosides. The PPT-type ginsenoside content in the xylem was relatively low, first exhibiting a rising trend, followed by a gradual decrease. The PPT ginsenoside content of the treated xylem accumulated rapidly, reaching its the maximum value on June 18, when it displayed an increase of 125.2% compared with the content in the control group. The PPT ginsenoside content in the control phloem exhibited a gradual decreasing trend. After cold stress treatment, the PPT ginsenoside concentrations were similar to those of the control group; however, the variation was greater. The maximum values were observed on June 18 and were a 52.1% higher than the values in the control group.

The PPT ginsenoside concentrations in the fibrous roots exhibited a gradual upward trend. After cold stress treatment, there was accumulation over a relatively short period, with significant differences from the control group. The maximum value was observed on June 18, showing an increase of 37.1% when compared with the value in the control group. The PPT-type ginsenoside concentration in the control stem did not change significantly during the flowering period. However, it first increased and then decreased following cold stress treatment. It reached its highest on June 22, representing a 59.4% increase when compared with the level in the control group. The levels decreased again on June 24.

The PPT-type ginsenoside content in the control leaves was the highest of any of the tissue parts, with no significant changes observed during the flowering period. After cold stress treatment, the PPT-type ginsenoside content in the leaves decreased. It was lower than that in the control group throughout the flowering period, and was significantly lower on June 24, decreasing to its minimum value on June 26, 57.9% lower than the control value. These results indicate that low-temperature conditions promoted the accumulation of PPT-type ginsenosides in the xylem and phloem. Short-term low temperatures promoted PPT-type ginsenoside accumulation in the fibrous roots and stems, and low temperatures inhibited PPT-type ginsenoside accumulation in the leaves. The total saponin concentrations and PPD ginsenosides showed similar trends in response to low temperatures. The synthesis and accumulation of PPT-type ginsenosides in the different tissues followed the order xylem > stem > leaf > phloem > fibrous roots.

### Changes in PPD/PPT Values in Different Tissues Under Cold Stress

The PPD/PPT ratios were between 2.00–2.51 and 1.47–1.89 in the fibrous roots and phloem tissues, indicating that the accumulation of PPD-type ginsenoside in the fibrous roots and phloem is faster than that of PPT-type ginsenoside. In the xylem and leaves, the PPD/PPT ratio is slightly less than 1, indicating that the accumulation rates of the two configurations of saponins in the xylem and leaves are similar, while the PPD/PPT ratio in the xylem is between 0.21 and 0.48, indicating that the accumulation of PPT-type ginsenosides in the xylem exceeds that of PPD-type ginsenosides. This indicates that the biosynthesis of ginsenosides has obvious tissue specificity, which is consistent with the results of previous studies (Zhang et al., 2014, 2018).

The PPD/PPT ratio in each of the tissues examined changed following cold stress treatment. The PPD/PPT ratio order of the treatment groups was fibrous roots > phloem > leaf > xylem > stem, which was slightly different from the order observed in the control groups (Table 3). The PPD/PPT ratio decreased in the xylem, phloem, and fibrous roots, indicating that the proportions of PPT ginsenosides increased in the xylem, phloem, and fibrous roots. Conversely, the PPD/PPT ratio increased in the leaves, indicating that the proportions of PPD ginsenosides increased in the leaves. In addition, the PPD/PPT ratio did not change significantly in the stem, potentially because it is involved in transduction but not in biosynthesis. The results indicate that, following cold stress treatment, the biosynthesis of PPT-type ginsenosides were promoted in the ginseng main roots (xylem and phloem) and fibrous roots, and PPD-type ginsenoside biosynthesis was promoted in the leaves.

**TABLE 3:** PPD/PPT ginsenoside content ratios in different ginseng tissues under cold stress during the flowering stage.

Date	Xylem		Phloem		Fibrous roots		Stem		Leaf	
	CK	Treatment	CK	Treatment	CK	Treatment	CK	Treatment	CK	Treatment
6.14	0.79	0.64	1.67	1.45	2.47	1.84	0.38	0.36	0.84	0.88
6.16	0.82	0.74	1.47	1.42	2.51	1.85	0.42	0.48	0.82	0.73
6.18	0.77	0.70	1.61	1.22	2.00	1.86	0.45	0.42	0.80	0.74
6.20	0.77	0.65	1.89	1.32	2.19	1.77	0.38	0.40	0.86	0.88
6.22	0.74	0.71	1.57	1.22	2.06	1.80	0.36	0.35	0.91	1.00
6.24	0.88	0.67	1.81	1.30	2.20	1.70	0.42	0.29	0.90	1.40
6.26	0.85	0.73	1.85	1.49	2.12	1.72	0.47	0.22	0.85	1.33
6.28	0.89	0.74	1.78	1.46	2.00	1.72	0.51	0.21	0.81	1.29

### Change of Malondialdehyde and Osmoregulatory Substance Content Under Cold Stress

**FIGURE 9:**
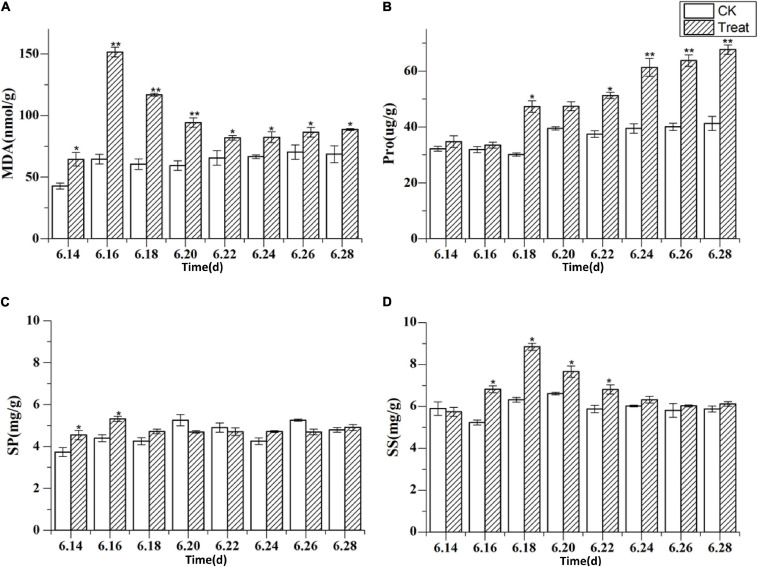
The content of malondialdehyde and osmoregulatory substances under cold stress. Panels **(A–D)** show the MDA, Pro, SP, and SS content under cold stress. The *X* axis represents the sampling time. *p < 0.05 and **p < 0.01, compared with control group. The data are expressed as the mean ± SD (*n* = 3).

MDA is the final product of membrane lipid peroxidation, and its content increases when a plant is subjected to oxidative stress. The concentrations of membranous peroxides and osmoregulatory substances following cold stress are shown in Figure 9. The MDA content increased rapidly and reached a peak on June 16, showing a significant increase of 134.6% compared with the MDA content in the control group (*p* < 0.01). The MDA content was maintained at levels higher than those in the control group throughout the treatment period. The Pro content peaked on June 28, showing a significant increase of 64.1% (*p* < 0.05) when compared to the levels in the control group. The highest SS content was observed on June 18, 39.7% higher than the level in the control group (*p* < 0.05). The effects on MDA, Pro, and SS were relatively obvious, while the effect on SP was less discernible, indicating that different osmotic substances have different functions in low-temperature regulation. There were also differences in the timing of activity.

### Change of Antioxidant Enzyme Activity Under Cold Stress

**FIGURE 10:**
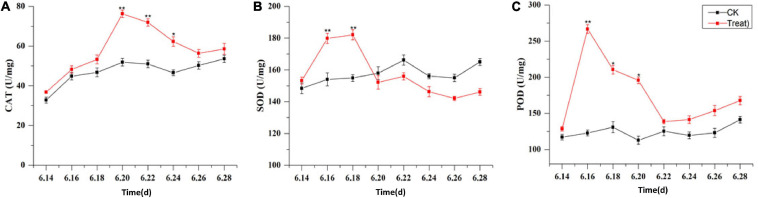
Effects of cold stress on antioxidant enzymes in ginseng leaves during the flowering stage. Panels **(A–C)** show the CAT, SOD, and POD content under cold stress. The *X* axis represents the sampling time. *p < 0.05 and **p < 0.01, compared with control group. The data are expressed as the mean ± SD (*n* = 3).

The effects of cold stress on antioxidant enzyme activity in the leaf are illustrated in Figure 10. Under cold stress, antioxidant enzyme activity, including SOD, POD, and CAT activity, increased initially and then decreased. SOD and POD responded rapidly to cold stress. Their maximum values were observed on June 18 and 16, respectively, and their activities were significantly higher (by 18.1 and 117.2%, respectively) than the levels in the control groups (*p* < 0.01). With the extension of the cold stress treatment period, the SOD and POD activities decreased gradually. The highest CAT activity was observed on June 20; it was significantly higher–41.1%–than the level in the control group (*p* < 0.01). With the prolongation of cold stress treatment, the CAT activity decreased gradually.

### Analysis of Key Enzymes Expression Under Cold Stress

**FIGURE 11:**
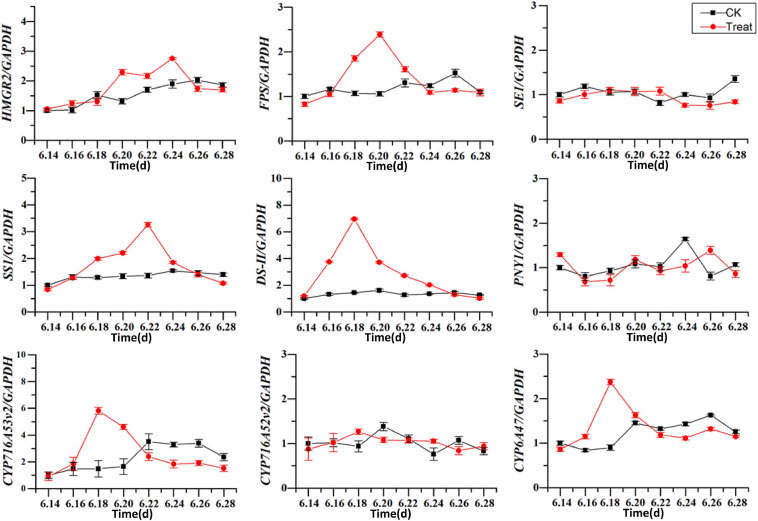
Effects of cold stress on the expression of key enzyme genes. The *X* axis represents the sampling time. *p < 0.05 and **p < 0.01, compared with control group. The data are expressed as the mean ± SD (*n* = 3).

The effects of cold stress on the expression of key enzyme genes associated with ginsenoside synthesis in the flowering stage are illustrated in Figure 11. *HMRG2*, *FPS*, *SS1*, *DS-II*, *CYP716A53v2*, and *CYP716A47* increased gradually in the control, and first increased and then decreased under low-temperature conditions. After cold stress treatment the *DS-II*, *CYP716A53v2*, and *CYP716A47* expressions reached their highest levels on June 18, and then decreased gradually. The maximum expression values were 4. 8-, 3. 9-, and 2.63-fold higher than the levels in the control group. The maximum level of *FPS* expression was recorded on June 20, and was 2.3-fold the level in the control group. The maximum level of *SS1* expression was observed on June 22, and was 1.9-fold the level in the control group. In addition, the maximum level of *HMGR2* expression was observed on June 24, and was 1.5-fold the level in the control group. These results indicate that different genes are expressed at different periods under cold stress treatment, with varying response times. In general, the levels of expression of key enzymes related to ginsenoside biosynthesis increased under short-term cold stress; however, under long-term cold stress, these gene expression levels decreased. Therefore, the secondary metabolic processes in pharmaceutical plants are influenced considerably at the transcriptional level by changes in temperature.

### Correlations Between Physiological Stress Indicators and Ginsenoside Content

Total ginsenoside content was significantly correlated with PPT-type ginsenosides and Re (*p* < 0.01), as well as with Rg1 and Rb1 (*p* < 0.05) (Table 4). Rg1 was significantly correlated with SE (*p* < 0.01) and with *DS-II*, *CYP716A52v2*, and *CYP716A53v2* (*p* < 0.05). In addition, Pro activity was significantly correlated with total ginsenoside, PPT-type ginsenoside, Re, and Rb1 (*p* < 0.01). Other physiological stress indicators were correlated with ginsenoside content; however, the correlations were not significant. In contrast, there were significant correlations between the expression levels of key enzymes in the synthesis pathway, antioxidant enzyme activity, and osmotic regulators. The results potentially indicated that antioxidant enzyme activities, osmoregulatory substances, and key enzyme gene expressions were jointly participated in ginseng resistance to cold stress. In addition, *SE*, *DS-II*, *CYP716A52v2*, *CYP716A53v2*, and Pro are potential cold stress response-related endogenous signaling molecules in ginseng.

**TABLE 4:** Correlation analysis among ginsenosides, physiological stress indicators, and secondary metabolism in plants under cold stress.

	Total	PPD	PPT	Rg1	Re	Rb1	*HMGR2*	*FPS*	*SS1*	*SE*	*DS-II*	*AS*	*52*	*53*	*47*	CAT	SOD	POD	Pro	MDA	SS	SP
Total	1	0.34	0.93**	0.77*	0.91**	0.74*	–0.49	0.24	0.52	0.54	0.57	–0.09	0.37	0.49	0.40	–0.35	0.70*	0.26	−0.86**	0.42	0.52	0.04
PPD		1	0.34	–0.21	0.58	0.37	–0.48	–0.57	0.06	–0.07	0.13	0.14	–0.27	–0.29	–0.37	–0.63	0.18	–0.06	–0.52	–0.16	–0.33	–0.14
PPT			1	0.76*	0.94**	0.88**	–0.51	0.13	0.52	0.67	0.56	–0.20	0.37	0.51	0.26	–0.30	0.71*	0.28	−0.95**	0.39	0.47	0.11
Rg1				1	0.55	0.66	–0.12	0.66	0.69	0.81**	0.71*	–0.47	0.77*	0.77*	0.67	0.17	0.78*	0.39	–0.59	0.66	0.86**	0.27
Re					1	0.78*	–0.65	–0.02	0.41	0.49	0.45	–0.07	0.23	0.24	0.19	–0.53	0.64	0.13	−0.93**	0.22	0.32	–0.06
Rb1						1	–0.23	0.10	0.36	0.78*	0.41	–0.13	0.36	0.47	0.13	0.04	0.52	0.10	−0.84**	0.20	0.40	–0.01
*HMGR2*							1	0.18	–0.47	–0.15	–0.44	0.17	0.16	–0.04	–0.08	0.81**	–0.53	–0.37	0.56	–0.32	–0.04	–0.24
*FPS*								1	0.38	0.58	0.32	–0.32	0.79*	0.35	0.95**	0.50	0.39	–0.02	0.15	0.34	0.91**	–0.07
*SS1*									1	0.61	0.99**	−0.80*	0.56	0.70	0.48	–0.19	0.94**	0.79*	–0.49	0.93**	0.60	0.71*
*SE*										1	0.60	–0.52	0.73*	0.58	0.57	0.32	0.69	0.21	–0.52	0.44	0.79*	0.15
*DS-II*											1	−0.77*	0.55	0.73	0.41	–0.21	0.94**	0.80*	–0.56	0.93**	0.57	0.71*
*AS*												1	–0.66	–0.45	–0.35	0.00	−0.75*	–0.60	0.18	−0.72*	–0.51	–0.66
*52*													1	0.38	0.78*	0.35	0.66	0.08	–0.17	0.43	0.92**	0.05
*53*														1	0.26	0.23	0.59	0.77*	–0.48	0.84**	0.47	0.70
*47*															1	0.22	0.54	–0.01	0.01	0.37	0.93	–0.11
CAT																1	–0.29	–0.24	0.45	–0.10	0.30	–0.12
SOD																	1	0.61	–0.65	0.80*	0.69	0.49
POD																		1	–0.38	0.92**	0.12	0.97**
Pro																			1	–0.38	–0.22	–0.22
MDA																				1	0.49	0.85**
SS																					1	0.03
SP																						1

Total: total saponin; HMGR: *HMGR2*; 52: *CYP716A52v2*; 53: *CYP716A53v2*; 47: *CYP716A47*. SOD, superoxide dismutase; POD, peroxidase; CAT, catalase; MDA, malondialdehyde; Pro, proline; SS, soluble sugar; SP, soluble protein. *p < 0.05, **p < 0.01.

## Discussion

The main active components of medicinal plants, such as the perennial herb *P. ginseng*, are secondary metabolites. Ginsenosides are the key bioactive compounds in ginseng and have diverse pharmacological applications. Ginsenoside concentrations are key criteria for evaluating the quality of ginseng medicinal materials (Kim et al., 2017). Both genetic and environmental factors influence ginsenoside biosynthesis, and appropriate cold stress levels could promote the accumulation of ginsenosides (Zhang et al., 2018). Cold stress is known to affect the growth and metabolism of plants (Jiang et al., 2016). In this study, short-term cold stress had no obvious effects on the dry or fresh weight of ginseng roots, while long-term cold stress significantly inhibited the accumulation of root dry and fresh weight.

The metabolic systems for scavenging ROS in plants are unbalanced under abiotic stress conditions, leading to the accumulation of ROS, which damages the plant membrane system. SOD, POD, and CAT are key components of the non-enzymatic antioxidant system in plants, and they can convert O^2–^ into H_2_O (Masood et al., 2006; Mhadhbi et al., 2011; Liu et al., 2016). Cold stress could promote the activities of SOD, POD, and CAT to a certain degree. In the present study, these three enzymes displayed higher activities when exposed to short-term cold stress, to help keep the plant functioning normally. This is consistent with previously published studies (Kaur et al., 2009). These three enzymes had the same response to cold stress, but the rates at which their enzyme activity increased, and the timing of their peak activity, differed. Under cold stress, the synergy of the different enzymes is needed to jointly combat oxidative damage in the plant. However, under long-term cold stress, the activities of the three enzymes were significantly reduced, probably because the ROS content exceeded the capacity of the antioxidant enzyme system of the plant. Thus, the antioxidant enzyme system could not prevent the damage caused by cold stress (Balestrasse et al., 2010).

MDA content is an indicator of the degree of lipid peroxidation in the cell membrane and can direct reflect the degree of cell membrane damage. In the present study, the MDA content increased under cold stress and peaked under short-term cold stress. It indicated that membrane lipid peroxidation increased under cold stress. The response times of the antioxidant enzymes were longer than that of MDA, indicating that plants can transmit signals to the antioxidant enzyme system by accumulating MDA, thereby stimulating the protective functions of the system. Osmoregulation is a critical physiological mechanism that facilitates plant adaptation to environmental stress. By adjusting the concentrations of osmoregulatory substances within the cells, plants can maintain cell turgor pressure and normal physiological processes. In the present study, the Pro, SS, and SP content increased during cold stress; the Pro content increased gradually as the duration of cold stress increased, which is consistent with observations made in a previous study. When *P. ginseng* was subjected to cold stress, the concentrations of osmoregulatory substances in the cells increased to facilitate the maintenance of osmotic balance in the plants (Lo’Ay and Elkhateeb, 2018).

*HMGR2*, *FPS*, *SS1*, *SE1*, *DS-II*, *PNY1*, *CYP716A52v2*, *CYP716A53v2*, and *CYP716A47* are the key genes that participate in the ginsenoside biosynthesis pathway, and their expression products are the corresponding enzymes. However, their high expression does not always indicate higher enzyme activity (Wang et al., 2012; Yang et al., 2018). In the present study, cold stress influenced the expression of the key genes of enzymes required for ginsenoside biosynthesis. The expression of *HMRG2*, *FPS*, *SS1*, *DS-II*, *CYP716A53v2*, and *CYP716A47* under short-term cold stress was significantly higher than in the control groups; however, under long-term cold stress, gene expression was reduced significantly, which is consistent with the findings of previous studies. This demonstrates that the key enzymes needed for ginsenosides biosynthesis first respond to cold stress at the transcriptional level and this, in turn, influences the biosynthesis and accumulation of ginsenosides.

When plants are exposed to cold stress, they can tolerate changes in the external environment by rapidly synthesizing secondary metabolites as a defense system (Cheng et al., 2018). Ginsenoside content is a key index for evaluating the quality of ginseng medicinal materials. Appropriate cold stress is conducive to the accumulation of ginsenosides. In the present study, ginsenoside content increased under short-term cold stress; however, the increase was inhibited significantly under long-term cold stress. Ginseng leaves are extremely sensitive to cold stress. This seriously inhibits ginsenoside biosynthesis in the leaves, causing the total saponin content in the leaves to drop sharply (Oh et al., 2014; You et al., 2015). The ginsenosides could be transferred to other tissues for storage via the xylem and phloem, since the saponins in the fibrous roots and stems increased by varying degrees under cold stress. This result is the same as that reported by Kim et al. (2014).

Recently, the relationship between active compositions and their bioactivities has attracted the attention of researchers. Previous study suggested that the composition of active ingredients is extremely important for the biological activity of ginseng (Shan et al., 2014). In addition, the PPD/PPT ratio is considered the key factor in determining the different biological activities of ginseng (Cho et al., 2017). PPD- and PPT-type ginsenosides play different roles in certain biological activities, and their compositional differences in ginseng are also a primary factor affecting the quality of the herb (Qu et al., 2009). Only when the PPD/PPT ratio in ginseng samples is within the optimal range can the quality of the ginseng medicinal materials be best reflected and its best medicinal value extracted. In this study, the PPD/PPT ratio in the xylem, phloem, and fibrous roots decreased under cold stress, and the proportion of PPT-type ginsenosides in the xylem, phloem, and fibrous roots increased. In contrast, the PPD/PPT ratio in the leaves increased. It indicates that cold stress promoted the biosynthesis of PPT-type ginsenosides in the main roots (xylem and phloem) and fibrous roots, as well as promoted the biosynthesis of PPD-type ginsenosides in the leaves. These results are consistent with the results of other studies on the accumulation of secondary metabolites in medicinal plants under cold stress (Klára et al., 2012; Dong et al., 2014; Carvajal et al., 2018; Rezaie et al., 2020).

Cold stress can influence secondary metabolic processes. For example, under certain temperatures, ginseng increases the activities of protective enzymes and the amounts of osmoprotective compounds to protect cells from oxidative stress. This enhances the biosynthesis of ginsenosides via the expression of key enzymes in the biosynthetic pathway and enhances secondary metabolite accumulation, in turn increasing the concentrations of certain ginsenosides. In the present study, the ginsenoside concentrations were significantly positively correlated with *SE* (*p* < 0.01), *DS-II*, *CYP716A52v2*, and *CYP716A53v2* (*p* < 0.05). In addition, the ginsenoside and Pro concentrations were significantly negatively correlated (*p* < 0.05). Antioxidant enzymes can promote or inhibit the expression and activities of key enzymes involved in ginsenoside secondary metabolism and influence the accumulation of ginsenosides. Therefore, *SE*, *DS-II*, *CYP716A52v2*, and *CYP716A53v2* could be the important candidate genes for ginsenoside biosynthesis in response to cold stress, and Pro could be exploited as an endogenous signaling molecule involved in ginseng responses to cold stress.

## Conclusion

According to the findings of the present study, under cold stress, *P. ginseng* activates its antioxidant enzyme system and accumulates osmoregulatory substances; however, cold stress also reduces the yield of *P. ginseng*. Short-term cold stress could promote the biosynthesis of ginsenosides significantly, and long-term cold stress could inhibit the biosynthesis of ginsenosides. In addition, short-term cold stress promoted PPT-type ginsenoside accumulation in the main roots (phloem and xylem) and fibrous roots, and PPD-type ginsenoside accumulation in the leaves. Furthermore, cold stress led to shifts in the PPD/PPT ratios in different tissues. Therefore, during the cultivation of *P. ginseng*, areas with lower average annual temperatures should be selected to facilitate the establishment of a balance between yield and quality. In industrial production processes, PPD/PPT ratios could be adjusted by low-temperature storage. The results of the present study could facilitate the exploitation of *P. ginseng* as a medicinal resource, through the manipulation of the concentrations of ginsenosides in different tissues, according to specific applications and requirements.

## Data Availability Statement

The original contributions presented in the study are included in the article/supplementary material, further inquiries can be directed to the corresponding author/s.

## Author Contributions

TZ and WQ conceived and designed the experiments. TZ performed most of the experiments, analyzed the data, and completed the first draft. CC, YC, QZ, and QL worked together with TZ to accomplish the experiments. All authors read and approved the manuscript.

## Conflict of Interest

The authors declare that the research was conducted in the absence of any commercial or financial relationships that could be construed as a potential conflict of interest.

## Abbreviations

CAT: catalase
MDA: malondialdehyde
CYP716A52v2: β-alanine C-28 hydroxylase
DS: dammarenediol synthase
HMGR: 3-hydroxy-3-methylglutaryl-CoA reductase
HPLC: high-performance liquid chromatography
Pro: proline
POD: peroxidase
PPD: protopanaxadiol
PPT: protopanaxatriol
ROS: reactive oxygen species
SOD: superoxide dismutase
SP: soluble protein
SS: soluble sugar
SS1: squalene synthetase
SE: squalene epoxidase.
